# Socio-emotionalcompetencies in teachers of educational institutions in the department of cordoba, acomparative study between men and women

**DOI:** 10.1192/j.eurpsy.2024.1265

**Published:** 2024-08-27

**Authors:** E. P. Ruiz Gonzalez, M. N. Muñoz Argel, A. M. Romero Otalvaro, M. G. Gracia Castañeda

**Affiliations:** ^1^ Universidad Pontificia Bolivariana; ^2^Universidad de Cordoba, Montería, Colombia

## Abstract

**Introduction:**

According to Bisquerra Alzina (2003),competencies are defined as a set of knowledge, capabilities, skills and attitudes, necessary to understand, express and regulate emotional phenomena appropriately and which are fundamental in the teaching profesion since they are closely related to students´performance and mental health.

**Objectives:**

compare socio-emotional skills in two groups of participants: female and male

**Methods:**

A non-experimental,cross-sectional design was proposed for this study. The scope of this research is descriptive, in the sense,that it seeks to establish measures in regard to specific variables. Sample (100 female and 100 male).

**Results:**

Results revealed that the evaluated teachers show average level of socio-emotional competencies, (Table 1).The highest scores were encountered in relation to the optimism competence. It suggests that teachers have the ability to obtain favorable balances from adverse situations presented in their daily lives.

**Table 1:** Distribution of socio-emotional competency levels in the professionals evaluated

	LOW %	MEDIUM %	HIGHT %
EMOTIONAL AWARENESS	19	80	1
SELF EFFICACY	32	66	2
EMOTIONAL REGULATION	17	81	2
EMOTIONAL EXPRESSION	6	85	9
PROSOCIALITY	6	85	9
ASSERTIVENESS	6	82	12
OPTIMISM	0	21	79
EMOTIONAL AUTONOMY	25	71	4
EMPATHY	8	85	7

Findings showed that there exists a statiscally significative difference (P=0,000) in the empathy and self-efficacy dimensions. Women obtained higher scores in these two abilities in regard to men. (Table 2). No differences were observed in the rest of the competences evaluated.

**Table 2:** Differences according to men and women

	FEMALE	MALE
SELF EFFICACY	1,78	1,61
EMPATHY	2,02	1,96

**Conclusions:**

Although teachers´s socio-emotional competences were classified in medium levels, it is necessary to implement an intervention design that allows to streghten those dimensions since they could improve not only the relationships with their students but also teachers´ mental health.

**Disclosure of Interest:**

None Declared

